# Terahertz Image Detection with the Improved Faster Region-Based Convolutional Neural Network

**DOI:** 10.3390/s18072327

**Published:** 2018-07-18

**Authors:** Jinsong Zhang, Wenjie Xing, Mengdao Xing, Guangcai Sun

**Affiliations:** 1 National Laboratory of Radar Signal Processing, Xidian University, Xi’an 710071, China; jszhang_1@stu.xidian.edu.cn (J.Z.); gcsun@xidian.edu.cn (G.S.); 2 Xi’an Gaoxin No.1 High School, Xi’an 710075, China; xwj2837mail@sina.com

**Keywords:** terahertz image detection, deep learning, transfer learning, threshold segmentation, Faster R-CNN

## Abstract

In recent years, terahertz imaging systems and techniques have been developed and have gradually become a leading frontier field. With the advantages of low radiation and clothing-penetrable, terahertz imaging technology has been widely used for the detection of concealed weapons or other contraband carried on personnel at airports and other secure locations. This paper aims to detect these concealed items with deep learning method for its well detection performance and real-time detection speed. Based on the analysis of the characteristics of terahertz images, an effective detection system is proposed in this paper. First, a lots of terahertz images are collected and labeled as the standard data format. Secondly, this paper establishes the terahertz classification dataset and proposes a classification method based on transfer learning. Then considering the special distribution of terahertz image, an improved faster region-based convolutional neural network (Faster R-CNN) method based on threshold segmentation is proposed for detecting human body and other objects independently. Finally, experimental results demonstrate the effectiveness and efficiency of the proposed method for terahertz image detection.

## 1. Introduction

In response to an increasing threat of terrorism, personnel surveillance at security checkpoints is becoming increasingly important [1,2]. Typical detection systems such as metal detectors for personnel and X-ray systems for hand-carried items are effective but also have a lot of shortcomings. Metal detectors can only detect metal targets, such as handguns and knives and cannot discriminate similar items. X-ray imaging systems can penetrate clothing barriers to image items concealed by common clothing. The disadvantages of X-ray systems are that their radiation is very high and they are very damaging to the human body. As a result of that, X-ray systems are only used to detect hand-carried items. The terahertz systems are also a typical imaging systems for personnel surveillance. Working at 30–300-GHz frequency band, terahertz system can also be called the millimeter wave system [3,4,5]. Terahertz systems are nonionizing and pose no known health hazard at moderate power levels. Higher frequency represents shorter wavelength (1–10 mm), which leads to higher resolution of terahertz image [4,5,6]. The current terahertz security imaging system can be divided into two categories: passive imaging and active imaging. Similar to the synthetic aperture radar (SAR), by synthesizing the real aperture into a larger virtual aperture [7,8,9,10], terahertz active imaging system could obtain clear human images which reveals the reflection characteristics of the concealed objects carried on human body. After getting the human image, it’s very important and meaningful to decide whether dangerous objects are carried or determine corresponding categories of these objects automatically. This paper focuses on terahertz active imaging for security applications and aims to realize the high-speed and high-accuracy detection of concealed items in terahertz images.

Constant false alarm rate (CFAR) is a commonly used detection method in SAR image detection [11]. In order to distinguish backgrounds and different targets, it mainly concentrates on analyzing the distribution characteristics of targets and clutters in the image by sliding windows [12]. Because of higher resolution and lower dimension, terahertz human images suggest more geometric features of the target than SAR images, as a result of that, CAFR detection method which focuses on target’s statistical characteristics makes no sense in terahertz image detection. Therefore, an effective method of feature extraction is very essential for terahertz detection. If the extracted abstract features contain sufficient information of the geometric structure and statistical characteristics of targets, the irrelevance between different targets, or the independence between backgrounds and targets can be maximized. For optical images, traditional image feature extraction methods can be divided into the following categories: Fourier transform [13], window Fourier transform (Gabor) [14], wavelet transform [15], least squares [16], boundary direction histogram [17], texture feature extraction based on Tamura texture feature [18] and so forth. Especially, over the last decade, great progress has been made in various visual identification tasks, largely based on the use of scale invariant feature transform (SIFT) [19] and histogram of oriented gradient (HOG) [20]. Although these feature extraction methods have many advantages, such as suppressing the effects of translation, rotation and illumination-changing for image, however, some factors like slow speed, high complexity and the need for artificial participation limit their application scopes [21,22].

With the development of these traditional methods, the artificial neural network (ANN) has gradually attracted peoples’ attention [23,24,25]. Extending from ANN, convolutional neural networks (CNN) were first proposed by LeCun et al. [26] in the 1990s, meanwhile they applied the stochastic gradient descent via back-propagation to train the CNN availably and achieved good recognition results but then it fell out of fashion with the rise in support of vector machine (SVM) [27]. SVM maps low dimensional data to higher dimensions and constructs a hyperplane based on nonlinear transformation of kernel function to achieve effective data classification in high-dimensional space. Compared with neural network, SVM method has a more solid mathematical theory foundation. It can effectively solve the problem of constructing high-dimensional data models under limited sample conditions and has the advantages of high generalization ability, easy to converge and dimension insensitivity. As a result, SVM has occupied the field of data classification and regression for a long time until a paper [28] published in 2012 broke the silence of neural network. Krizhevsky et al. trained a large, deep CNN (Alexnet) to classify 1.2 million high-resolution optical images into 1000 different classes, which attained the highest classification accuracy at that time [28]. CNN have lots of prior knowledge to compensate for all data and their generalization ability could be improved by adjusting the model’s depth and width. The Alexnet is made up of 5 convolutional layers and 3 fully connected layers and contains a number of unusual features which reduces its training time and improve its classification performance. The most critical parts of this network are rectified linear units (ReLUs) and dropout strategy. ReLUs improve the nonlinear mapping ability of this network and avoid the problem of vanishing gradient. Dropout reduces complex co-adaptations, as a result of that, Alexnet could learn more robustness features. At the same time, the enhancement of GPU computing power also provides a hardware foundation for such a large network and so many parameters. Since the Alexnet was proposed, there has emerged a large number of papers to expose deeper and more adaptable networks (e.g., [29,30]). Nowadays, classifying optical or natural images with CNN has also been matured gradually, as a matter of course, if these deep learning methods can be applied to terahertz images, the detection performance for concealed items will be greatly improved.

When training a new neural network with different data or different tasks, it’s an expensive operation to begin training with a group of random weights, for example, long training time or not well convergence. Considering that most data or tasks are related, we can speed up the model’s learning efficiency by fine-tuning with a well-trained model and this sensible optimization strategy could be called transfer learning. A scientific survey [31] (by Pan et al., 2009) divided transfer learning into the following aspects: unsupervised transfer learning, transductive transfer learning and domain adaptation/generalization and so forth. Donahue et al. studied a semi-supervised deep convolution method for multitask learning which is the first application of transfer learning in CNN. The trained model associated with this learning method can also be considered as a supervised pre-training phase and the feature extracted from this model is called the caffe feature. Nowadays, caffe has become a very widely used open source framework for deep learning with its excellent GPU acceleration capability and simple training process. In this paper, we also adopt this training framework to train an effective classification network, at the same time, the training strategy of transfer learning has also been used in view of structural similarity between the optical image and the terahertz human image.

It is noteworthy that we have been discussing the application of deep learning in image classification till now. Compared with identifying target’s category, object detection is also a very challenging and difficult task since the detection task doesn’t only need to identify a variety of goals for different categories in the same image but also need to obtain the accurate ground truth of corresponding targets. The traditional object detection method for optical images can be summarized as the following steps: (i) getting a rough detection box by sliding multi scale and different size window in the image; (ii) extracting features from images based on a classic feature extractor, for example, SIFT, HOG, LBP et al.; (iii) identifying targets’ categories in the detection box by importing the extracted features into a trained feature classifier, for example, SVM. As we mentioned above, these object detection methods may not work very well when backgrounds are complex or different from each other and the reason can be explained as that these image features extracted by traditional methods have a poor generalization ability, result in negative adaption to actual conditions. However, for that the brain neurons can extract more advanced and more essential characteristics of objects, humans can identify different objects accurately even under bad environmental conditions. Analogously, the convolution neural network based on bionics also has good feature representation ability which is why it has developed so quickly in recent years.

Since the CNN has shown excellent ability in image classification, extending it to image detection also becomes one of the pop problems in the field of image processing. There are two main problems with image detection: localizing objects accurately and training a high-capacity classification model with small-sample labeled data; fortunately, Girshick et al. gave out a satisfactory answer of named regions with CNN features (R-CNN) [32] by bridging the gap between image classification and object detection. When processing an image, R-CNN first generates around 2000 different region proposals, extracts a fixed-length feature vector from each proposal with well-trained feature-extracted model, then classifies each region with category-specific linear SVMs. The Region-based CNN achieves excellent object detection accuracy; however, this method has notable drawbacks: 1. using multi-stage pipeline to train a model; 2. sacrificing a lot of time and space resources; 3. the detection speed is too slow. Considering the reduction of the training and testing time, spatial pyramid pooling networks (SPPnet) [33] were proposed to speed up R-CNN by sharing computation and convolutional features. Although SPPnet accelerates R-CNN by 10 to 100 times at test time, it’s still a multi-stage pipeline like R-CNN. Therefore, an improved R-CNN (Fast R-CNN) was proposed by Girshick et al. which is a single-stage training algorithm that jointly learns to classify object proposals and refine their locations and this method nearly achieves real-time detection when ignoring the time spent on region proposals. Based on the Fast R-CNN, Ren et al. [34] replaced traditional region proposal methods with a fully convolutional network named region proposal network (RPN) and the RPN can simultaneously predict object bounds and object probability. Merging RPN and Fast R-CNN into a single network, Faster R-CNN has a frame rate of 5 fps on a GPU and there is no doubt that is a great progress for object detection. Besides that, YOLO [35], SSD [36] and R-FCN [37] et al., are all the most popular method for image detection.

However, these mentioned methods are designed for optical image datasets for example, PASCAL VOC 2007 [38] and MS COCO [39] and terahertz images are different from these open datasets since radar images and optical images have different imaging mechanisms. Specifically, terahertz image reflects object’s electromagnetic characteristics and these characteristics vary with observation angle, target structure and material factors. Fortunately, the high working frequency of the imaging system is much closer to the optical spectrum, as a result, terahertz images of security inspection still have a lot of similar geometric features to optical images. For example, the large areas of knives’ metal materials are all represented as a series of regular bright blocks and the brightness of a knife-handle is very low in the terahertz and optical images. In this paper, we attempt to transfer these classification methods and detection methods of optical images to terahertz images. Dataset plays a very important role in deep learning. In other words, the more data, the better performance. So, we collect lots of terahertz images and label these images as the standard data format of VOC2007. Then, we establish the Terahertz Classification Dataset and discriminate a few objects in terahertz image with the classification method based on transfer learning of optical features. After solving the classification problem, considering the particularity of terahertz images, we propose an improved Faster R-CNN method based on threshold segmentation for the human body and other objects' detection.

This paper begins in Section 2 with the showing results of terahertz imaging and the introduction of Terahertz Human Dataset. In Section 3, the classification method and detection method of concealed items are explained. The experimental results and corresponding analysis are also descripted in this section. Section 4 discusses some problems and Section 5 concludes this paper.

## 2. Dataset

In this section, we first introduce terahertz imaging theory and some related imaging results, then a little terahertz images which include different targets and multiple projection angles are showed to understand the spatial distribution of terahertz image. Since the detection method based on deep learning requires a large number of images as training samples, we establish two datasets: the Terahertz Human Dataset for object detection and the Terahertz Classification Dataset for object classification.

### 2.1. Imaging Description

**Figure 1:**
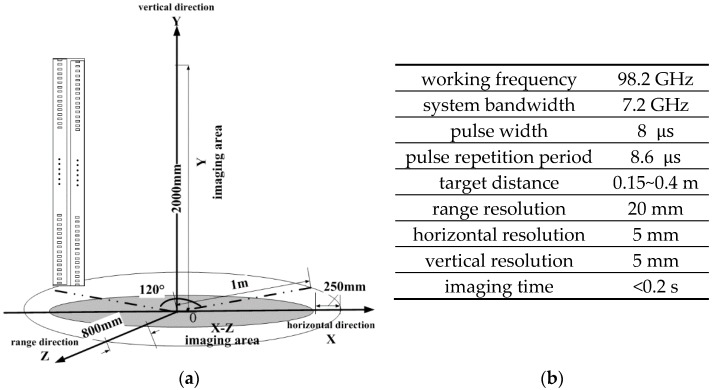
Terahertz imaging description. (**a**) The structure diagram of terahertz imaging system. (**b**) The working parameters of the imaging system, like working frequency, resolution and imaging time.

The terahertz imaging system works by synthesizing the small and real aperture into a larger virtual aperture to obtain human images [8,10]. And the best imaging way is covering the human body with an authentic and holonomic antenna aperture, however, this method requires a large number of echo acquisition units and it’s too expensive. As a result of that, the imaging system replaces this high cost way with a holographic linear array of sequentially switched transmitter receivers scanned quickly over a large aperture to actively illuminate the target [1,2], in other words, the system will transmit coherent signals at different positions and receive corresponding echoes. As Figure 1a shows, the system includes two working machines: frontal imaging and back imaging. Each machine has four transmitting and receiving antennas. When the system begins to work, these beam scanning antennas will scan in horizontal direction keeping a fixed rotation speed. As a result of that, beam can quickly cover human body. Partial working parameters are shown in Figure 1b. This terahertz imaging system is very similar to SAR at imaging principle. Advantages of this technique include: (1) near real-time operation; (2) high-resolution; (3) computer reconstruction allows focusing at any single depth; and (4) large aperture (full-body field of view) [3].

The system images from original echo with fast back-projection. This method is a SAR imaging algorithm based on range frequency domain and azimuth time domain. Firstly, range compression is performed in the same way as the basic RD imaging algorithm, then the accumulation curve in the range compression signal is calculated and the values on the curve are coherently accumulated. Finally, the azimuth processing is completed to obtain the target image. We can attain 12 high resolution human images from different positions by scan imaging.

**Figure 2:**
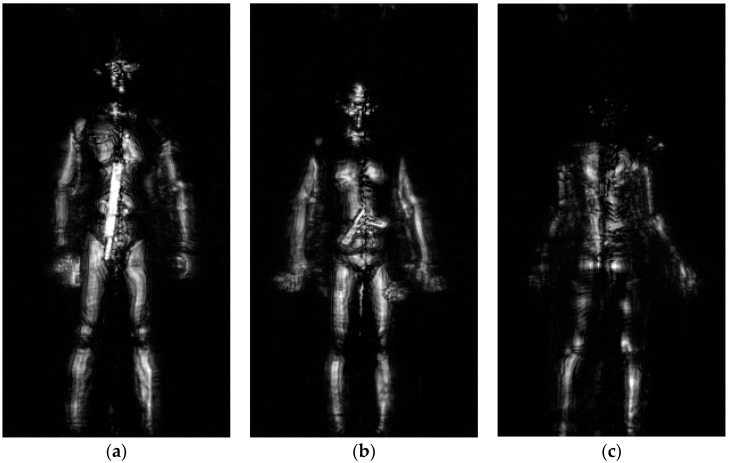
Terahertz human imaging results. (**a**) The image is imaging from frontal angles and the man in this image carried a long knife. (**b**) The image is imaging from frontal images and the man carried a metal simulation handgun. (**c**) The image is imaging from back angles and the man didn’t carry any targets.

The resolution of terahertz image is approximately 5 mm. A few imaging results are showed in Figure 2. In order to save storage space, these images are saved in JPEG format and the scale of each image is fixed 380 × 200 pixels. With eliminating the environmental clutter, the background of these images is clear and the human body also presents a clear profile. These concealed objects in the human image are clearly visible for that terahertz imaging system has a high resolution and is readily penetrate common clothing material. The thickness of the clothes is uneven, so the intensity of human body also represents uneven. To test the imaging performance of concealed items, when collecting these images, several typical targets including knives, handguns, phones and bottles have been carried and concealed on the human body with clothes, for example, sweater, down jacket and wool coat. Because the millimeter-waves are penetrate common clothing material and are reflected from the human body and any concealed items, especially are sensitive to metal materials, the intensity of interesting objects, for example, the knife in Figure 2a and the handgun in Figure 2b is higher than human body and corresponding contour features of different objects are very clear. So, compared with easily ignoring these objects in optical human images, we can detect these concealed items in terahertz images intuitively.

### 2.2. Terahertz Human Dataset

Object detection in terahertz images refers to localizing objects of interest (e.g., handguns, knives) on the human body and predicting their categories. Datasets have played an important role in data-driven research in recent years. So, we have collected about 30,000 terahertz images of several different kinds of dangerous and common targets according to the actual needs of security inspection, then these images are labeled as the standard of VOC 2007 [38], to be exact, we record the ground truth and objective category in each terahertz image. Naturally, we name the set of these annotated images as Terahertz Human Dataset. The number and scale of different objects are listed in in Table 1 to understand the dataset. There are 26,505 images in the Terahertz Human Dataset and each image takes the human body as a reference. In addition, there are about 1 k~4 k unequal numbers of targets, including knife, handgun, bottle and phone. The reason for choosing these goals is that they are easily distinguishable from each other and are more typical in the field of security. We also list these objects’ scale according to the standard [wmin, wmax, hmin, hmax] and the corresponding meaning is the object’s minimum width, maximum width, minimum height and maximum height in all images. Take the object of the knife as an example, its scale is [3, 129, 8, 113], considering terahertz image’s resolution, the knife’s real scale is [1.5, 64.5, 4, 56.5] millimeter. On the foundation of the Terahertz Human Dataset, in Section 3 and Section 4, we describe our terahertz target classification method and target detection method based on deep learning. In order to ensure that the training data and test data distributions approximately match, we randomly select 80% of the original images as the training set, 10% as validation set and 10% as the testing set.

### 2.3. Terahertz Classification Dataset

**Figure 3:**
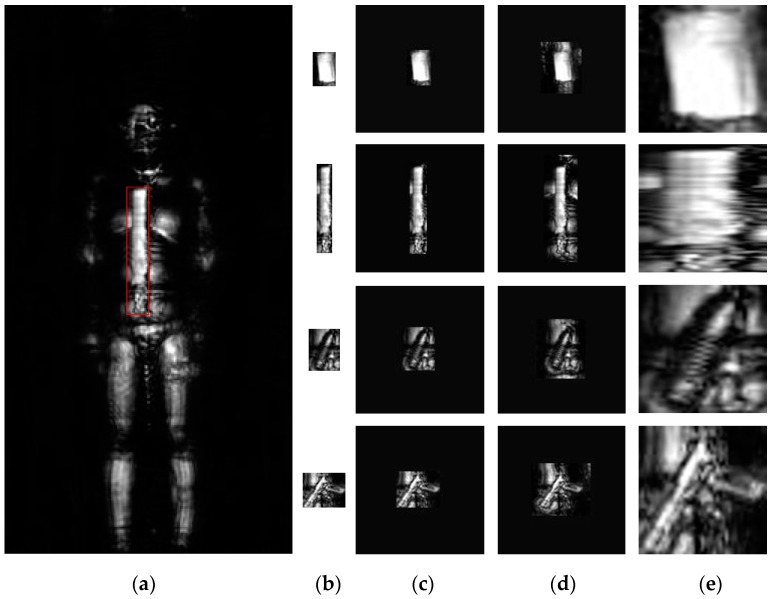
Different transformations of terahertz images. (**a**) An example of the original annotated terahertz image; (**b**) The original cutting images of interesting object; (**c**) Filling with the image mean; (**d**) Cutting with context and filling with image mean; (**e**) Wrapping.

Object classification is different from object detection in the field of computer vision, because the purpose of classification is judging the object’s category, meanwhile, the classification model takes fixed scale images as network input (e.g., 227×227 pixels). So, on the foundation of building a Terahertz Human Dataset for object detection, we also need to collect enough target images for training a good classification model. In this paper, we cut out interesting targets from terahertz human images directly. As Figure 3 shows, there are usually three cutting strategy and choosing a more optional way is very important for that affects the classification performance to a great extent.

Figure 3a shows an original annotated terahertz human image with a bounding box which records object’s location information by rule and line. A natural processing way is cutting this object image as the rule of bounding box and filling this image to the required image size with mean pixels for its effect will be removed by the subtract-mean procedure of the classification model. Figure 3b,c show this cutting processing and filling results. Another transformation approach is cutting with context and filling with image mean [32]. This isotropic scaling method considers additional context around the original bounding boxes and makes use of object’s background information, as a result of that, this method performs well in the classification task. The size of context padding (p) is defined as a border size around the original object bounding box and we show the results of p=8 pixels in Figure 3d [32]. Except these two methods, wrapping is also a common transformation way for its flexible operability. Nevertheless, as Figure 3e shows, this anisotropy scaling method changes object’s contour and geometric features, so the classification result of this method is not very well. In this paper, we use the method of cutting with context and filling with image mean to 227×227 pixels. The object composition of Terahertz Classification Dataset keeps the same with Terahertz Human Dataset, as listed in Table 1, when ignoring the object scale.

## 3. Proposed Method

The object detection method based on region proposal is consists of two branches: one network is to locate the object’s bounding box, another is to determinate corresponding object’s category [34]. As a result of too few annotated training samples, these detection methods should not initialize the model’s weight randomly and the common way is to use a well-trained classification model to initialize the detection network. On the foundation of well initialization, the network’s weight also needs to be tuned for the task of object detection. There are lots well pre-trained models and these models are all trained by optical images, unfortunately, there are not exoteric or popular similar terahertz classification model until now. Unlike optical images, terahertz image reflects target’s geometrical features and electromagnetic characteristics simultaneously. So, the terahertz images are different from optical images and they are informative but these images’ resolution is lower than that of optical images, if we still use the optical pre-trained model to initialize the terahertz detection network directly, the target detection result could not be good for these differences. Considering the correlations of the optical image and terahertz image of the same object, we first train a classification model in Section 3.1 by transfer learning with the feature of optical image. Then we propose an improved Faster R-CNN method in Section 3.2 for terahertz object detection. When training this detection model, the network parameters is initialized by transfer learning with the well-trained terahertz classification model. After training the classification model and detection model, we also analyze the experimental results to assess the performance of mentioned methods.

### 3.1. Terahertz Classification Model

In this section, we fist introduce a modified CNN classification model for terahertz image classification, then considering the similarities between terahertz images and optical images, a few training strategies are proposed to train a well network. The experimental analysis demonstrates the effectiveness

#### 3.1.1. Architecture of CNN

**Figure 4:**
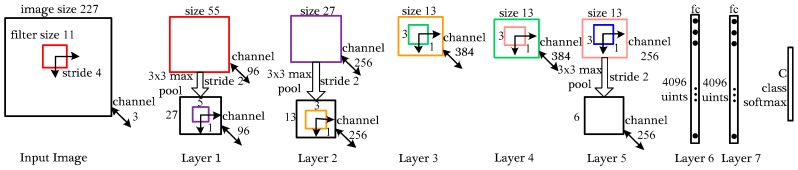
An illustration of used CNN architecture. The network’s input is 154,587 (227×227×3) dimensional and the number of neurons in the network’s remaining layers is given by 290,440-186,624-64,896-64,896-43,264-4096-4096-4. The numbers of convolutional parameters and fully connected parameters in the network is given by 34,944-614,656-885,120-1,327,488-884,992-37,752,832-16,777,216-16,384.

After establishing the Terahertz Classification Dataset in Section 2.3, we use the modified AlexNet [28] to classify the terahertz objects. As Figure 4 shows, the network contains eight computing layers: five convolutional networks and three followed fully-connected networks. The overall network takes the fixed-size 227×227 pixels image as input and the output of the last fully-connected layer is fed to a C-way softmax which produces a distribution over the C class labels. In this paper, the number of labels C equals four (knife, handgun, bottle and phone).

The first convolutional layer takes the original image as input and filters corresponding image with 96 kernels of size 11×11×3 with a stride of 4 pixels. After convolution, pooling layer subsamples the 55×55×96 feature maps with 3×3 max-pooling with a stride of 2 pixels. The pooling layer doesn’t change map’s channel but decreases map’s size to 27×27×96 pixels conversely. The second layer works like the first layer with convolution and pooling and the size of acquired feature maps is 13×13×256. The convolution process of layer 3, layer 4 and layer 5 keep the same size of filtering kernels, meanwhile, the existence of 1 pixel stride also keeps the size of the feature map unchanged. It’s worth noting that the pooling layer in the layer 5 decreases the final feature map to 6×6×256. The fully-connected layers fc6 and fc7 have 4096 neurons each and these neurons are all connected to upstream network nodes. As Figure 4 shows, the parameters of the fully-connected network are far more than the parameters of the convolutional network. Too much network parameters will lead to over-fitting which could weaken the network’s generalization ability overwhelmingly. So, the pooling layer in this network could decrease lots of parameters and perform well in the classification task.

To improve the ability of high-dimension representation, the network takes a nonlinearity function to active each neuron in the feature maps. Considering the convergence speed and prediction precision, the non-saturating nonlinearity f(x)=max(0,x) which was referred as Rectified Linear Units (ReLUs) is used in this convolutional network. In the process of repeated and iterative training, the network’s output error between real labels and predictions results is propagated layer by layer by the back-propagation algorithm, so the loss function is very important for that could affect the update accuracy of these layer’s weights. The loss function of softmax layer we used is defined as follows:

(1) $$l\left(\theta \right)=-\frac{1}{m}\left[\sum_{i=1}^{m}\sum_{j=1}^{k}1\left\{y^{i}=j\right\}\log\frac{e^{\theta_{j}^{T}x\left(i\right)}}{\sum_{l=1}^{k}e^{\theta_{l}^{T}x\left(i\right)}}\right]+\frac{\lambda }{2}\sum_{i=1}^{m}\sum_{n=1}^{k}\theta_{i\;n}^{2}\;$$

where m is the number of training samples for once iteration, k is the number of labels, θ is the network’s weights which need to be tuned and λ is the regularization coefficient. Besides that, the indicator function 1{·} in Equation (1) takes on a value 1 if its logic arguments are true and 0 otherwise.

Optimization algorithm for decreasing the training loss plays a key role in the task of image classification. While traditional stochastic gradient descent (SGD) algorithm remains a very popular optimization strategy in the field of deep learning, training models with it can sometimes be slow. The SGD with momentum is used in this paper for that can accelerate learning, especially in the face of high curvature, small but consistent gradients. This optimization method is summarized in Algorithm 1 [40].

**Algorithm 1.** Stochastic gradient descent (SGD) with momentum.**Require:** Initial parameter θ, initial velocity v.**Require:** Learning rate ε, momentum parameter α. **while** do stopping criterion not met **do** Sample a minibatch of m images from the training set with corresponding label $y^{\left(i\right)}$
 Compute gradient estimate: $g:=\;\frac{1}{m}\nabla_{\theta }\sum_{i}L\left(f\left(x^{\left(i\right)};\theta \right),y^{\left(i\right)}\right)$
 Compute velocity update: v:=αv−εg
 Apply update: θ:= θ+v

**end while**


#### 3.1.2. Transfer Learning

A well-trained convolutional neural network could extract the critical characteristic of different objects efficiently. In order to obtain excellent generalization ability, the network based on statics theory specifies that the training data and testing data belong to the similar parameter distribution. As Section 2.3 indicates, there are only a few thousands of terahertz images for classification, even some of these images are not clear enough to distinguish from others. As a result of that, if the terahertz network is trained from zero (random initialization), the training results may not be well (over-fitting or not convergence). Fortunately, the Transfer Learning is the answer we are pursuing which could assist the training process of the target task with the learned information of the source task. There are usually two ways of implementing of transfer learning: one way is the feature extraction, which takes the well-trained model from source task as a feature extractor and relearns the last few increased layers without changing the original network parameters; another way is the fine-tuning, which adds a few random-initialization layers to the pre-trained networks, moreover, the weights of original layers will be updated in a small learning rate [31]. In detail, when the quantity of training samples is too little or the distribution of target task is different from the source task, the transfer learning tunes the bottom layers and removes the deeper and specific layers [34].

**Figure 5:**
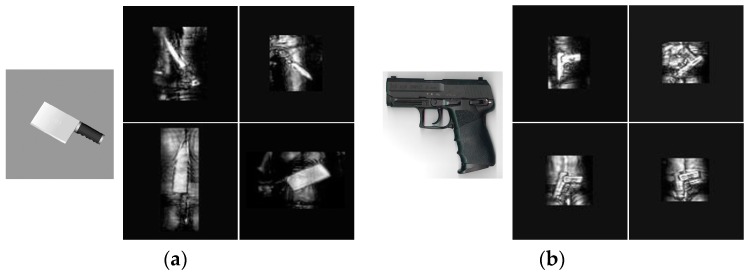
Similarity comparison. The contrast images of optics and terahertz system of similar objects. (**a**) Knife. Left column shows an optical image of kitchen knife and the right column shows four different types of terahertz knife images and they are assumed as the same object. (**b**) Handgun. Left column image is the optical image of gun and right columns show four terahertz images which are imaging from different angles.

In this paper, the terahertz classification tasks are different from the optical tasks. The resolution of terahertz images is much lower than corresponding optical images and the terahertz imaging system is very sensitive of human body and metal materials, so the distinguishability of terahertz images is worse than that of optical images. However, as for the same object, there are little similarities between terahertz images and optical images. For example, the large area of knives’ metal materials in Figure 5a are all represented as a series of regular bright blocks, the contour difference of knives and corresponding backgrounds is very significant and the brightness of knife-handle is very low in the terahertz and optical images. As for Figure 5b, both the optical and terahertz images show the right-angle structure of the handgun. So intuitively, we first assume that the object features in the terahertz images and optical images belong to the similar distribution, then the terahertz classification model is trained with the mentioned fine-tuning strategy, finally we will prove the validity of the hypothesis distribution by experiments.

**Figure 6:**
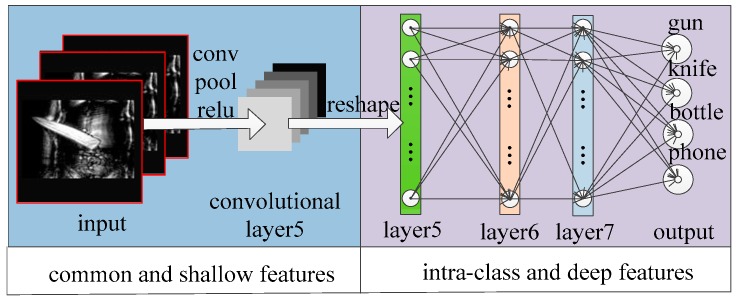
The interpretation of features extractions for different layers.

The most popular classification models are all based on optical images which has three color (RGB) channels [28,29,30], however, the terahertz images are consisted of the only one gray channel. In order to make use of the mature model we mentioned above, as the left side of Figure 6, the terahertz images are reproduced from the one channel to three identical channels.

Besides that, according to Girshick’s work [32], the ahead convolutional layers could extract common and shallow features (Figure 6 left) and the later fully-connected layers focus on maximizing the difference characteristics between different objects (Figure 6 right). Based on the well-trained classification model, this paper mainly adjusts the weights of fully-connected layers (fc6, fc7, softmax-layer) and the parameters of convolutional layers are adjusted at a little speed. Correspondingly, the basic learning rate of the classification network is initialized as 10 × 10^−3^ and the actual learning rate of convolution layers and fully-connected layers are the results of the basic learning rate multiplies different factors, naturally, the different combination of these factors decides the different learning ability of this network. We show a few combinations of different multiplicative factors in Table 2.

We propose four different training strategies in Table 2, for that they can make use of the characteristic difference between the convolutional layers and fully-connected layers. The first row represents these training strategies and the first column represents different layers, including convolutional layers, fc6 layer, fc7 layer and softmax-layer. Such as fine-tuning fc7, when training the network, the learning rate of layers before fc7 is the result of that the basic learning rate multiplies the corresponding multiplicative factors and both the factors of convolutional layers and fc6-layer equal 1.0 here, so the learning rates keep the same with basic learning rate. Naturally, the learning rate of layers including fc7 layer and layers behind fc-7 layer are the results of that the basic learning rate multiplies 10. As a result of that, the network could learn the features extracted by fc7 layer and layers behind fc7 quickly.

#### 3.1.3. Experiment Analysis

We use the Terahertz Classification Dataset in Section 2.3 to train the model. And this model was trained with the SGD algorithm [40] with a basic learning rate of 0.001, a batch size of 128 examples, momentum of 0.9 and weight decay of 0.0005. The weights without pre-trained layers were initialized from a zero-mean Gaussian distribution with standard deviation 0.01 and corresponding neurons biases are set to 0. Except that, we also adjusted the basic learning rate manually with dividing the rate by 10 when the validation error stopped improving with the current learning rate. We stopped training after 85 epochs which took around 26 h on a single Tesla K20c GPU.

**Figure 7:**
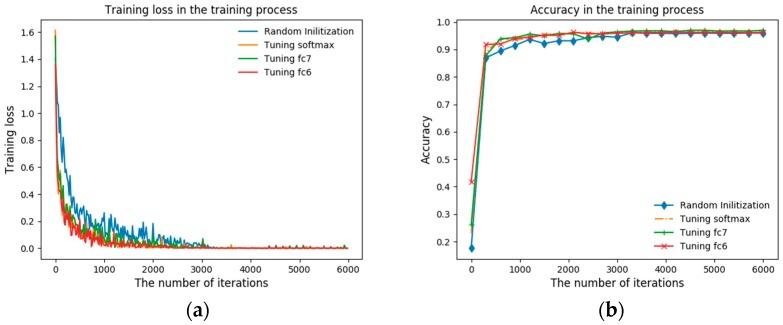
The variation of training loss and testing accuracy in the training process. (**a**) Training loss in the training process. (**b**) Testing accuracy in the training process.

In this section, we train the classification model with the four-learning strategy (random initialization, fine-tuning softmax, fine-tuning fc7, fine-tuning fc6) in Table 2. The Figure 7 reveals the training details and Figure 7a shows the variation of training loss in the training process. The training loss curve of random initialization is high and tends to be volatile. Compared with random initialization, the role of the fine-tuning is very obvious for that the amplitude of the three fine-tuning training loss curves, especially the loss of fine-tuning fc6, becomes smaller and smoother. At the same time, the three fine-tuning curves have converged in the 2500 iteration and perform very well than training by the random initialization (converged in the 3500th iteration). It is worth noting that the final convergence error of these four training strategies have almost the same value. Corresponding to training loss, the testing accuracy in the training process is as Figure 7b shows and accuracy curves of the fine-tuning also perform much better than random training with a faster convergence speed. What’s also important is that all these curves are increasing monotonically before convergence and there is no over-fitting in the training process, this classification results proves the effectiveness of the model in this paper.

Except that, we also list the best classification accuracy of these four learning strategies in Table 3. The random initialization has a classification accuracy of 95.98% of four typical terahertz objects. The fine-tuning learning methods improve this accuracy by 0.7%~1.0%, especially the fine-tuning fc7 turns out best ultimately. So, in the training process of terahertz classification, the fine-tuning training methods based on different fully-connected layers that we proposed in this paper could not only shorten the training time by nearly 30% but also improve the classification performance of the convolutional network. The main reason why fine-tuning could get a better result than random initialization can be explained as follows: terahertz images and optical images have a different degree of similarity in imaging mechanism and geometric characteristics, therefore, the automatic feature extraction methods based on convolutional network could make use of these similarities and remedy the problem of the lack of terahertz images. As a result of that, the classification performance of this model is guaranteed to be effective.

### 3.2. Terahertz Image Detection

For terahertz human image detection, we should not only determine the object’s category and position but also need to locate the human body in original images for subsequent manual checks. There are two main type deep-learning methods of image detection: one is based on direct regression which could detect the category and location with feature maps that are extracted by well-trained convolutional network, this method run fast and achieve real-time detection but the detection precision still need to be improved. Another method is the “two-step” detection method based on region proposal: first, the most promising object areas are extracted from the features maps of original whole image, then the object classification network would determine the type of these objects. One of the most typical representative method based on region proposal is Faster R-CNN [34], which could accurately detect the location and category of objects and this method is a little slower than the detection method based on direct regression for its two-step strategy, fortunately, the running speed can also achieve satisfactory results of 5 fps processing speed. Except that, the detection method based on deep learning processes human and other objects simultaneously despite the human body overlapping with other objects. As a result of that, there will be contradictions when the network is used for classification: when detecting concealed objects, the human body will become the background noise; when locating human body, these objects will also affect the geometry features of human body. So, different detection methods are needed to determine human body and other objects. In this section, we first analyzed the amplitude distribution of terahertz human image, then an improved Faster R-CNN method based on threshold segmentation was proposed to deal with the conflict between human body and objects, finally the analyses of experimental results show the method’s effectiveness.

#### 3.2.1. Threshold Segmentation

**Figure 8:**
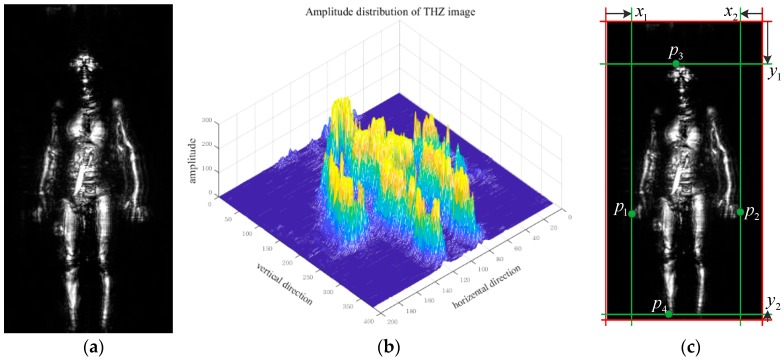
The amplitude distribution of human body in a terahertz image. (**a**) An original terahertz image. (**b**) 3-D amplitude image consistent with Figure 7a. (**c**) Threshold segmentation method based on bounding-slipping.

As Figure 8a,b show, the background clutter is low and the intensity (amplitude) of the human body is much higher than the background in a terahertz image, so an effective method is to use the threshold segmentation to locate the human body in the original image. The traditional pixel-based segmentation method determines that the current processing pixel belongs to which object and the clear outline of targets could be attained directly. However, what is needed to do is to get the bounding box in the task of human detection. This paper proposes a threshold segmentation method based on bounding-slipping to locate the human body in terahertz image. The algorithm principle is as Figure 8c shows and the algorithms steps are as follows:

Firstly, the decision threshold T is given as the following principles:

(2) $$T=\frac{1}{MN}\sum_{m=1}^{M}\sum_{n=1}^{N}g\left(P_{n}\right)\;$$

where M is the number of training images, g(⋅) is the amplitude of corresponding point, $p_{n}$ is the point with the maximal amplitude in each sides of bounding box of human body and N is the number of $p_{n}$, N=4 here. After getting T, as Figure 8c shows, the deciding-line (the 4 red lines in Figure 8c) slips in 4 directions which include left-to-right, right-to-left, up-to-down and down-to-up. When the maximal amplitude of terahertz image in line l meets the conditions:

(3) max(g(l))>T 

the line l is decided as the bounding line (the 4 green lines in Figure 8c) and corresponding point is decided as the bounding point ($p_{1}$, $p_{2}$, $p_{3}$ and $p_{4}$). At the same time, the location of human body is decided as the rectangular area which is surrounded by the bounding lines. The threshold segmentation method based on bounding-slipping could locate human body quickly despite the effect of other sensitive objects. In the next section, this segmentation method is fused with Faster R-CNN for realizing an ordered and unified terahertz image detection system.

#### 3.2.2. The Improved Faster R-CNN

**Figure 9:**
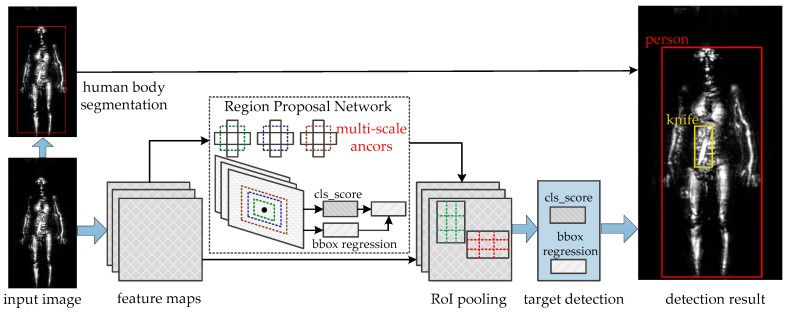
The network structure of the improved Faster R-CNN algorithm.

The Faster R-CNN object detection system is composed of two parallel modules [34]: Region Proposal Network (RPN) and Fast-RCNN. RPN is a deep fully convolutional network that proposes regions and Fast-RCNN detectors could use the proposed regions to detection objects. Besides that, we also add the threshold segmentation method to this network for locating human body. The entire system is a single, unified network for terahertz object detection (Figure 9).

The RPN takes an image (of any size) as input and outputs a set of rectangular object proposals, each with an object score. Specifically, we first use the network in Section 3.1 to extract the convolutional features from original images. Then, a small network which takes a n×n spatial window of the convolutional feature maps as input is mapped to a lower-dimensional features. This feature is fed into two sibling fully-connected layers: a box-regression layer (*reg*) and a box-classification layer (*cls*), which could decide the box as a set of object classes or background. At each sliding-window location, we set k reference boxes (anchors) associated with a scale and aspect ratio to improve the detection accuracy. We keep the same anchors of Faster R-CNN: 3 scales and 3 aspect ratios, yielding k=9 at each sliding position. The function loss of RPN is defined as [33]:

(4) $$L\left(\left\{p_{i}\right\},\left\{t_{i}\right\}\right)=\frac{1}{N_{cls}}\sum_{i}L_{cls}\left(p_{i},p_{i}^{*}\right)+\lambda \frac{1}{N_{reg}}\sum_{i}p_{i}^{*}L_{reg}\left(t_{i},t_{i}^{*}\right)\;$$

here, i is the index of an anchor and $p_{i}$ is the predicted probability of anchor i being an object. $t_{i}$ is a vector representing coordinates of the predicted bounding box and $t_{i}^{*}$ is that of the ground-truth. $N_{cls}$ is the size of minibatch and $N_{reg}$ is the number of anchor locations. The parameterizations of coordinates for bounding box regression following [34]:

(5) $$\begin{array}{l} t_{x}=(x-x_{a})/w_{a},\;t_{y}=\left(y-y_{a}\right)/h_{a},\\ t_{w}=\log\left(w/w_{a}\right),\;t_{h}=\log\left(h/h_{a}\right),\\ t_{x}^{*}=\left(x^{*}-x_{a}\right)/w_{a},\;t_{y}^{*}=\left(y^{*}-y_{a}\right)/h_{a},\\ t_{w}^{*}=\log\left(w^{*}/w_{a}\right),\;t_{h}^{*}=\log\left(h^{*}/h_{a}\right). \end{array}\;$$

where x,  y , w and h denotes the box’s center coordinates and its width and height. Variables x, $\;x_{a}$ and $x^{*}$ are for the predicted box, anchor box and ground-truth box respectively.

Besides adding the human body location method to the Faster R-CNN, another improvement that we’ve come up with is the fine-tuning tragedy for training this end-to-end network. The paper [34] initialized the convolutional layers with the pre-trained model for ImageNet [41] classification. What is different is that we initialize the original convolutional layers with the well-trained model for terahertz object classification in Section 3.1. All new layers in the network are randomly initialized by drawing weights from a zero-mean Gaussian distribution with standard deviation 0.01. We use a momentum of 0.9 and a weight decay of 0.0005 to optimize the loss function. And other parameters are all the same with the paper.

#### 3.2.3. Experimental Analysis

In order to validate the performance of our algorithm, a few popular networks which include YOLOv2, SSD, R-FCN and FRCNN are all trained with the terahertz dataset [34,35,36,37]. Both YOLO and SSD belong to the detection method based on regression. YOLO runs very fast but the detection accuracy of small object performs very poor. SSD imbibes advantages of both YOLO and RPN and considers the detection speed and detection accuracy. The Region-based fully convolutional net (R-FCN) [37] could balance the location invariance for classification and the location variance for detection by position sensitive score map. We also compare the detection performance of Faster R-CNN (FRCNN) [34] and the Improved Faster R-CNN (IFRCNN). The testing result are shown in Table 4:

The average precision (AP) is a detection measure which combines the classification accuracy and location accuracy for each object. *mAP* is the mean AP for all objects. As for Table 4, there are three important points: firstly, for human body detection, IFRCNN performs very well compared to other methods with the AP of 98.75%, which almost achieves to the level of human observation. Secondly, for knife detection, the R-FCN performs the best in the first four methods and the IFRCNN also have a slight improvement with 2%~18% AP. Thirdly, for handgun, bottle and phone detection, especially for *mAP*, the IFRCNN have a different degree of improvement than other detection methods.

Compared with other methods, the proposed IFRCNN performs well in the terahertz image detection. In order to further analyze the detection result of IFRCNN, we list the missing alarm and false alarm of these targets of IFRCNN in Table 5. Missing alarm is the ratio of the number of not-detected targets to the number of real targets and false alarm is the ratio of the number of misclassification targets, for example, judging a knife as a handgun, to the real number of this kind of targets. The lower missing alarm and false alarm, the better detection precision of this target. From Table 5, we can see that the person has the lowest missing alarm and the lowest false alarm, which indicates that the proposed method could detect and locate human body at a very high precision. Compared with handgun, the knife has a lower missing alarm and high false alarm and that illustrates this proposed IFRCNN method missing the knife with a small probability and judging the knife as other targets with a higher probability. The bottle and phone can be analyzed as this comparison methods.

**Figure 10:**
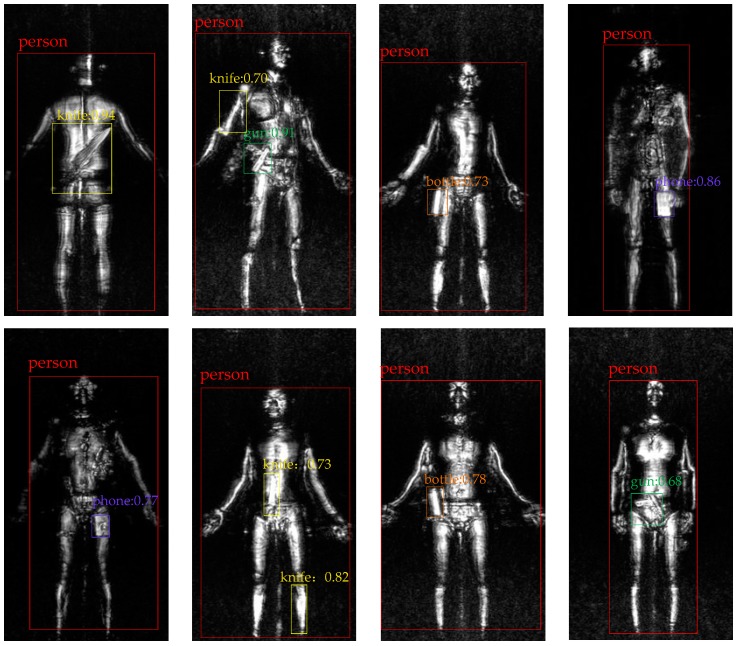
Selected detection results with IFRCNN on terahertz image dataset.

We also select a few detection results of terahertz image in Figure 10. Each predicted box is associated with a category label and a softmax score in [0, 1] except that ‘person’ are only labeled with category. A score threshold of 0.6 is used to display these images and the same color represents the same object in all image. The running time for obtaining these results is near 100 ms per image with IFRCNN.

## 4. Discussion

In general, the reason why IFRCNN performs better than other methods in terahertz image detection lies in the carefully designed human detection method and the fine-tuning method based on transfer learning. Firstly, the FRCNN could extract features from the whole image and then classify objects with these features. This method works well in detection of optical image, however, when the method is used for terahertz image detection, an important prior knowledge is ignored with that the human body represents the largest size and the relatively fixed location in terahertz image. At the same time, compared with SAR image, terahertz image is not affected by the multiplicative nature of the speckle, therefore, the background of terahertz image represents clearer and an intuitive detection method is to use the threshold segmentation to locate the human body. Testing results showed that our IFRCNN method performs well in the human body detection of terahertz image. Another important point is the fine-tuning training strategy. For IFRCNN, we adopt this strategy twice, the first time is to initialize the terahertz classification model with the well-trained optical classification model and another is to initialize the convolutional parameters with the terahertz classification model. We didn’t only make use of the similarity between terahertz image and optical image to classify the terahertz object but also transferred this similarity to the training of terahertz image detection. Intuitively and factually, this training strategy works well in the task of terahertz image detection.

Table 4 and Figure 10 shows a few detection results of IFRCNN and most of these results are in line with corresponding ground truth. It’s worth noting that the detection method often misjudges the limbs of human body as a knife and this phenomenon leads to a higher false alarm probability. This is not the faultiness of the detection method, for that people often confuse them because of the low-resolution of terahertz image (compared with optical image). In order to solve this problem in the future, a suggestion method is to use the pedestrian recognition to segment the human body with other objects and it requires further research.

## 5. Conclusions

In this paper, we proposed an improved detection method based on deep learning for terahertz image detection. The detection method based on deep learning has become a hot field of computer vision for its excellent performance and simple adjustment of network parameter. The deep learning is based on statistics and its relationship with data is very important. In other words, the more data, the better performance of the detection method. Therefore, in the task of terahertz image detection, we collected a lots of terahertz images and labeled these image as the standard of imaging condition and human observation. On the basis of that, the Terahertz Human Dataset and Classification Dataset was established. Then, considering the similarity between terahertz image and optical image, we proposed the classification method based on transfer learning of optical features. Inspired by the fixed distribution of human body in terahertz image, we also proposed the threshold segmentation method based on bounding-slipping. Furthermore, we coalesced this method and Faster R-CNN into a uniform system for terahertz detection. Besides that, we also used the well-trained terahertz classification model to initialize this detection system. Compared to other popular detection methods, the method is much competitive in terms of the detection accuracy. More importantly, the proposed method runs very fast which makes our method more practical for real applications.

## Figures and Tables

**Table 1:** The composition of Terahertz Human Dataset.

Type	Quantity	Scale
person	26,505	[25, 289, 28, 363]
knife	4494	[3, 129, 8, 113]
handgun	2591	[10, 67,1, 63]
bottle	1747	[9, 60, 16, 83]
phone	1190	[8, 48, 11, 84]

**Table 2:** Multiplicative factors of different strategies.

	Random Initialization	Fine-Tuning Output	Fine-Tuning fc7	Fine-Tuning fc6
convolutional-layers	1.0	1.0	1.0	1.0
fc6 layer	1.0	1.0	1.0	10
fc7 layer	1.0	1.0	10	10
softmax-layer	1.0	10	10	10

**Table 3:** Testing accuracy of well-training models.

Strategy	Random Initialization	Fine-Tuning Output	Fine-Tuning fc7	Fine-Tuning fc6
accuracy	95.98%	96.68%	96.98%	96.75%

**Table 4:** Detection average precision (%) with different algorithm.

	YOLOv2	SSD	R-FCN	FRCNN	IFRCNN
person	85.34	90.78	91.06	90.43	98.75
knife	67.90	81.08	83.71	80.36	85.44
handgun	67.45	69.21	68.18	67.32	70.06
bottle	30.28	44.64	42.46	38.89	46.72
phone	34.84	45.05	46.47	45.53	47.18
*mAP*	57.16	66.15	66.38	64.51	69.70

**Table 5:** Missing alarm and false alarm of IFRCNN.

	Missing Alarm	False Alarm
person	1.25%	3.47%
knife	10.14%	18.53%
handgun	30.32%	8.06%
bottle	58.02%	15.73%
phone	48.17%	20.90%
